# Interactions between microRNA-200 family and Sestrin proteins in endometrial cancer cell lines and their significance to anoikis

**DOI:** 10.1007/s11010-019-03547-2

**Published:** 2019-05-09

**Authors:** Joanna Kozak, Paulina Wdowiak, Ryszard Maciejewski, Anna Torres

**Affiliations:** Department of Normal Anatomy, Medical University of Lublin, 20-090 Lublin, Poland

**Keywords:** Endometrial cancer, Sestrin, miR-141, miR-200a, Luciferase reporter assay, Anoikis

## Abstract

In the present study, we intend to determine whether Sestrin proteins 1, 2, and 3 (SESN1-3) are targets of microRNA-200 family (miR-200) in endometrial cancer (EC) Ishikawa, AN3CA, KLE, and RL 95-2 cell lines and to investigate how these potential interactions influence anoikis resistance of EC cell lines. The luciferase reporter assay, qRT-PCR, and western blotting assays were used to verify whether SESN1-3 are direct targets of miR-200. Moreover, the anoikis assay and transient transfections of miR-200 mimics or inhibitors into EC cell lines were performed to evaluate the modulatory role of miR-200 and SESN proteins on anoikis resistance. We demonstrated that SESN2 protein is a direct target of mir-141 in KLE and RL-95-2 EC cell lines and the functional interaction of miR-141 and SESN2 protein has a downstream effect on anoikis resistance and SESN2 expression level in Ishikawa and AN3CA cell lines. Moreover, we have shown that SESN3 protein is a direct target of miR-200b, miR-200c, and miR-429 in Ishikawa, AN3CA, and KLE cell lines. Our results show that manipulation of miR-200b, miR-200c, and miR-429 expression patterns also has an influence on anoikis resistance in EC cell lines. In conclusion, we identified new interactions between miR-200 and the oxidative stress response SESN proteins that affect anoikis resistance in human EC cells.

## Introduction

Endometrial cancer (EC) remains a major cause of female reproductive tract malignancy [1]. Early diagnosed EC carries a favorable prognosis with high survival rate [2]. However, the prognosis for the patients with late-stage disease is poor with low survival rate [3]. The poor prognosis could be related to progression of endometrial cancer that is characterized by greater invasion of myometrium and lymph node metastasis [4]. In this regard, we took a closer look at anoikis, the cell death induced by cell detachment from extracellular matrix. Anoikis seems to be an important step in the tumor metastatic cascade that could be regulated by reactive oxygen species (ROS), oxidative stress, or hypoxia [5]. We hypothesized that Sestrin (SESN) proteins could play some regulatory role of anoikis in endometrial cancer.

SESN1 to 3 belong to conserved stress-responsive proteins regulated by p53 and forkhead transcription factor (FOXO) [6]. SESN1 (initially known as PA26) was discovered in mammalian cells under hypoxia, radiation condition, and some chemotherapeutic agents’ treatment [7]. SESN1 is under control of p53 tumor suppressor and is implicated in cell growth regulation by modulation of mTOR (mechanistic target of rapamycin) signaling pathway [8]. SESN1 functions as an antioxidant by the regeneration of overoxidized peroxiredoxins [9]. Another member of Sestrin protein family, SESN2 is expressed under different stress conditions as genotoxic stress, oxidative stress, mitochondrial dysfunction, or endoplasmic reticulum stress [10]. SESN2 shares significant homology to SESN1 and both of them belong to growth arrest and DNA damage-inducible family protein (GADD). However, regulation of SESN2 expression by p53 occurs in response to DNA damage but not oxidative stress [11]. SESN2 expression is also regulated by hypoxia inducible factor (HIF)-1α [12]. SESN2 the same as SESN1 activated upon genotoxic and oxidative stress has the potency to inhibit cell growth by mTOR signaling pathway suppression through an AMPK-dependent mechanism [8, 13, 14]. Moreover, the protective function of SESN2 in oxidative stress is connected with nuclear factor erythroid 2-related factor 2 (Nrf2) and Kelch-like ECH-associated protein 1 (Keap1) pathway with assistance of p62 protein [15]. SESN2 promotes p62-dependent autophagic degradation of Keap1 and activates Nfr2. As a consequence, Nrf2 can induce the transcription of antioxidant enzymes in response to oxidative stress [16, 17]. The last member of Sestrin proteins family is SESN3. Although SESN3 shares gene sequence homology with SESN1 and 2 [6], its expression is regulated by FOXO transcriptional factors but not by p53 [18]. SESN3 expression level is elevated in vigorously metabolizing cells that consume a large amount of oxygen and produce reactive oxygen species (ROS) [19]. It is thought that SESN3 is the main regulator of ROS downstream of Akt signaling pathway and FOXO transcriptional factors [19]. In summary, all SESN proteins play a protective role against oxidative stress by reducing the intracellular ROS level, but do this in different signaling pathways [6].

A number of studies have shown that microRNAs (miRNAs) are potent regulators of cellular signaling pathway connected with oxidative stress and hypoxia [20–22]. Based on those findings, we hypothesized that SESN proteins could be also regulated by miRNAs. miRNAs negatively regulate gene expression on post-transcriptional level through binding to 3′UTR sequence of target mRNA, and thus often cause mRNA destabilization and degradation [23]. In our study, we focused on miRNA-200 family which includes miRNA-200a, miRNA-200b, miRNA-200c, miRNA-141, and miRNA-429 [24]. The members of miRNA-200 family are divided into two functional groups based on seed region homology. Functional group I consists of miRNA-200b, miRNA-200c, and miRNA-429 and functional group II consists of miRNA-200a and miRNA-141 [25]. The miR-200 family deregulation in endometrial cancer is associated with cellular processes such as epithelial-to-mesenchymal transition (EMT), anoikis resistance, or migration and invasion [26–28].

To expand the knowledge on the potential role of SESN proteins in endometrial cancer anoikis resistance, we investigated the expression of all SESNs known in humans so far by measuring mRNA and protein level in four gynecological cancer cell lines commonly used in experimental research: Ishikawa, AN3CA, KLE, and RL 95-2. In addition, we set out to identify the potential relationship between up-regulated miR-200 family and SESN proteins using the Luciferase reporter experiments. To determine whether these interactions are correlated with anoikis resistance of EC cell lines, we performed transient transections of miR-200 mimics and inhibitors and anoikis assay into EC cell lines. Consistent with the anoikis experimental results, we investigated the potential effects of miR-200 family members on SESN protein expression levels.

## Materials and methods

### Cell culture

Endometrial carcinoma cell lines KLE (ATCC CRL1622TM), AN3CA (ATCC HTB111TM), RL-95-2 (ATCC CRL1671TM), and human fibroblast (ATCC CRL-2106) were purchased from ATCC (American Type Culture Collection, Manassas, VA, USA). Ishikawa was purchased from Sigma-Aldrich (St. Louis, MO, USA). AN3CA and fibroblast cell lines were maintained in MEM (Gibco, Thermo Fisher Scientific, Waltham, MA, USA) supplemented with 10% fetal bovine serum (FBS) (Gibco, Thermo Fisher Scientific, Waltham, MA, USA) and 2% penicillin/streptomycin (PAN-Biotech GmbH, Aidenbach, Germany). The Ishikawa cell line was maintained in MEM supplemented with 5% FBS and 2% penicillin/streptomycin. KLE cell line was maintained in DMEM (Gibco, Thermo Fisher Scientific, Waltham, MA, USA) supplemented with 10% FBS and 2% penicillin/streptomycin. RL-95-2 cell line was maintained in DMEM: F12 (Gibco, Thermo Fisher Scientific, Waltham, MA, USA) supplemented with 10% FBS, 2% penicillin/streptomycin, and 0.005 mg/mL insulin (Sigma-Aldrich, St. Louis, MO, USA). All cells were grown in a humidified chamber at 37 °C in 5% CO_2_ atmosphere.

### Protein extraction and western blot analysis

The total protein was extracted by using RIPA lysis buffer (Sigma-Aldrich, St Louis, MO, USA) supplemented with protease inhibitors cocktails (Sigma-Aldrich, St Louis, MO, USA). After 30-min incubation on ice, the lysates were centrifuged at 10,000×*g* for 10 min, and supernatant was collected for experiments. The total protein concentration was measured using Bradford reagent (Sigma-Aldrich, St Louis, MO, USA). Protein lysates (10 µg) were resolved on denaturating gels with 10% sodium dodecyl sulfate–polyacrylamide (SDS-PAGE) (XCell SureLock™ Mini-Cell Electrophoresis System, Thermo Fisher Scientific, Waltham, MA, USA) and were transferred onto nitrocellulose membrane (iBlot Western Blotting system, Thermo Fisher Scientific, Waltham, MA, USA). For fluorescence detection, membranes were blocked in 5% non-fat milk in PBS for 1 h at 4 °C and were probed overnight at 4 °C with the following primary antibodies 1:500 dilution: anti- SESN1, anti- SESN2 (Sigma-Aldrich, St Louis, MO, USA), anti- SESN3 and 1:1000 dilution of anti-β-actin or 1:2000 dilution of anti-GAPDH (Cell Signaling Technology, Beverly, MA, Abcam, Cambridge, MA, USA). After the 1-h incubation with secondary antibodies IRDye 800 CW (1:10 000 dilution), the results were visualized by using Odyssey Infrared Imaging System (LI-COR Bioscience, Lincoln, NE, USA). Quantitation was performed by comparing the Integrated Intensity values that were automatically calculated by Odyssey software. Four replications were performed, and the values are shown as mean ± SD.

### RNA isolation and quality control

RNA isolation from cell lines was performed using mirVanaPARIS Kit (Thermo Fisher Scientific, Waltham, MA, USA) according to the manufacturer’s protocol. Concentration and purity of RNA was measured using spectrophotometry (Biophotometer with Hellma TrayCell, Eppendorf, Hamburg, Germany). 260/280 ratio of all RNA samples ranged between 1.8 and 2.2. All samples were stored at − 80 °C. RNA integrity was checked using Agilent Bioanalyser 2100 (Agilent Technologies Inc., Santa Clara, CA, USA). RIN values of RNA ranged between 6 and 8.6. Samples with RIN ≥ 6 were used for downstream applications.

### Quantitative real-time amplification (qRT-PCR) of mRNA

To analyze SESN1, SESN2, and SESN3 expression, mRNA was retrotranscribed with High Capacity cDNA Reverse Transcription Kit (Thermo Fisher Scientific, Waltham, MA, USA), followed by qPCR with specific primers according to the manufacturer’s protocol. All RT reactions were carried out in triplicates in Mastercycler ep gradient S (Eppendorf, Hamburg, Germany) and stored in − 20 °C. All qPCR reactions were performed in triplicates in the Viia7 detection system (Thermo Fisher Scientific, Waltham, MA, USA). The comparative Ct method was used to calculate relative expression of mRNA compared with UBC expression.

### Luciferase reporter experiments

In order to verify the specific interaction between miR-200 family and SESN proteins family, the co-transfection experiments were performed. On the first day of experiments, the cell lines were seeded to yield 80% of confluence at the time of transfection as follows: 10,000 cells/well for Ishikawa cell line, 16,000 cells/well for AN3CA cell line, 70,000 cells/well for RL-95-2 cell line, and 30,000 cells/well for KLE cell line. On the second day, all GoClone reporter constructs and microRNA were prepared. MicroRNA mimics miR-200a, miR-200b, miR-200c, miR-141, miR-429, and miR-NC were dilated to working concentration of 20 nM according to the manufacturer’s protocol (Active Motif, Carlsbad, CA, US). The transfection mixtures were made with OptiMEM serum free media (Gibco, Thermo Fisher Scientific, Waltham, MA, USA) and DharmaFect Duo transfection reagent (Active Motif, Carlsbad, CA, US). The mimics of miR-200b, miR-200c, miR-429, and miR-NC were co-transfected with 30 ng/µL pLightSwitch_3′UTR reporter vector (Active Motif, Carlsbad, CA, US) containing the 3′UTR sequence of SESN1 gene or SESN3 gene in all the tested EC cell lines. The mimics of miR-200a, miR-141, and miR-NC were co-transfected with 30 ng/µL pLightSwitch_3′UTR reporter vector containing the 3′UTR sequence of SESN2 gene in all the tested EC cell lines. Twenty-four hours following the transfection with microRNA mimics, 100ul LightSwitch Assay Solution (Active Motif, Carlsbad, CA, US) was added to each well and each plate was incubated for 30 min in room temperature to evoke luciferase reporter signal. Luminescence signal was recorded on VICTOR X4 multimode plate reader (Perkin- Elmer, Waltham, MA, US). The luciferase activity of the cells that were transfected with microRNA mimics was represented as the percentage of activity relative to that of the cells that were transfected with miR-NC. To control the non-specific effect associated with our experimental condition, we used pLightSwitch_Random 3′UTR control vector and pLightSwitch_ACTB 3′UTR control vector. The empty pLightSwitch_3′UTR reporter vector served as positive control for the transfection efficiency. All experiments were performed in triplicates.

### Anoikis assay and cells transfection

For the anoikis assay (CtyoSelect 96-Well Anoikis Assay; Cell Biolabs, Inc. San Diego, CA, USA), the EC cell lines were suspended in antibiotic-free regular growth medium and then seeded in triplicate in 96-well anchorage-resistant plate in the following densities: Ishikawa 20,000 cells/well, AN3CA 20,000 cells/well, KLE 40,000 cells/well, and RL 95-2 70,000 cells/well. Subsequently, cells were transfected with microRNA mimics miR-200a, miR-200b, miR-200c, miR-141, miR-429, and miR-NC at a final concentration of 20 nM per well using DharmaFect DUO transfection reagent. After incubation for 24 h at 37 °C in a humidified incubator with 5% CO_2_, the live cells were detected with Calcein AM. The working concentration of Calcein AM was prepared according to the manufacturer’s protocol and 1 µL was added to each well. The plate was incubated at 37 °C for 1 h. Fluorescence was measured at excitation wavelength 485 nm and emission wavelength 515 nm using a multimode plate reader VICTOR X4. Ishikawa, AN3CA, and KLE cell lines were also assayed using 96-well anchorage-resistant plate and microRNA inhibitors (Exiqon, Vedbaek, Denmark). The cells were prepared and seeded as described above. Next, the cells were transfected with microRNA inhibitors i-miR-200b, i-miR-429, and microRNA inhibitors cocktail i-miR-141 and i-miR-200a; i-miR-200b and i-miR-200c and i-miR-NC at a final concentration of 50 nM per well using DharmaFect DUO transfection reagent. 24 h after transfection, the live cells were detected with Calcein AM according to the manufacturer’s protocol. All experiments were performed in triplicates, and the values are shown as mean ± SD.

### Data analysis

qRT–PCR data analysis and Luciferase reporter experiments data analysis were performed using MS Excel and GraphPad Prism 6 software. The mean and SD were calculated using MS Excel. Comparisons between two independent groups were performed using Student’s t test. Statistical significance was determined by p value of less than 0.05. Statistical analyses were performed using GraphPad Prism 6 software.

## Results

### Discordance between SESN1, SESN2, and SESN3 mRNAs and proteins level in endometrial cancer cell lines

**Fig. 1:**
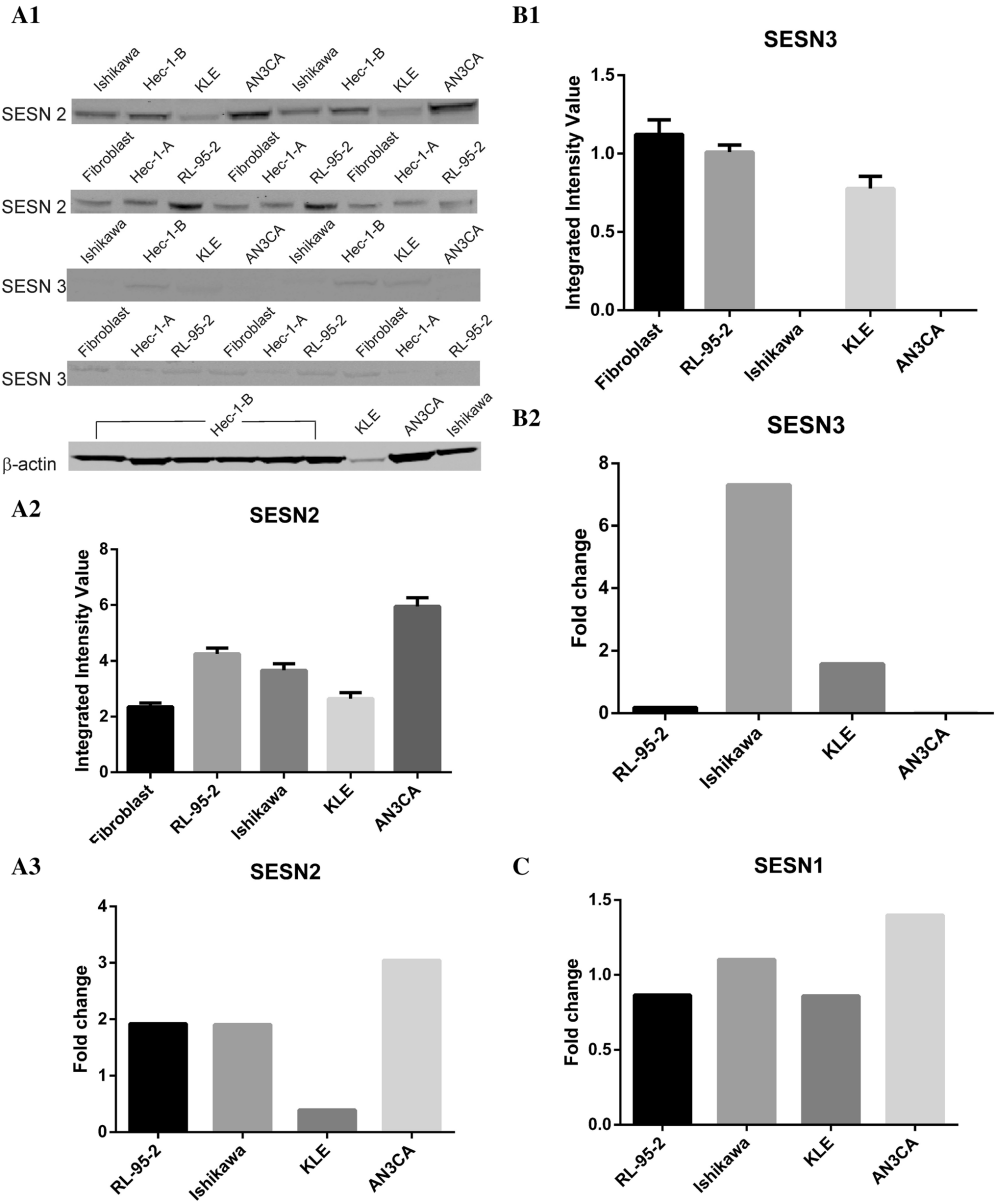
Profile of SESN1, SESN2, and SESN3 protein expression in human fibroblast and EC cell lines as assayed by Western blot and qRT-PCR analysis. **a1** Representative western blotting showing SESN2 expression was strong in AN3CA and RL-95-2 cell lines, moderate in the Ishikawa cell line, while it was faint in fibroblast and KLE cell lines. Representative western blotting showing SESN3 expression was faint in the KLE, RL-95-2, and fibroblast cell lines, while it was not detected in Ishikawa and AN3CA cell lines. β-actin was used as a loading control. **a2** Scanning with Odyssey Infrared Imaging System revealed different fluorescence intensity expressed as Integrated Intensity Value that correspond to SESN2 protein band of each analyzed cell line. Data are represented as mean ± SD. **a3** Relative expression of SESN2 mRNA in human fibroblast and EC cell lines proved higher expression in RL-95-2, Ishikawa, and AN3CA cell lines than in the KLE cell line. Data are normalized to UBC and relative to human fibroblast cell line. The fold change value less than 1 indicates down-regulation of target mRNA. **b1** Scanning with Odyssey Infrared Imaging System revealed different fluorescence intensity expressed as Integrated Intensity Value that correspond to SESN3 protein band each analyzed cell line. Data are represented as mean ± SD. **b2** Relative expression of SESN3 mRNA in human fibroblast and EC cell lines showed higher expression in Ishikawa and KLE cell lines than in RL-95-2 and AN3CA cell lines. Data are normalized to UBC and relative to human fibroblast cell line. The fold change value less than 1 indicates down-regulation of target mRNA. **c** The differential expression of SESN1 in EC cell lines. qRT–PCR assay showed that SESN1 is up-regulated in Ishikawa and AN3CA cell lines, whereas it is down-regulated in RL-95-2 and KLE cell lines. Data are normalized to UBC and relative to human fibroblast cell line. The fold change value less than 1 indicates down-regulation of target mRNA

The control cell line, human fibroblast and the Ishikawa, KLE, AN3CA, and RL-95-2 human EC cell lines were selected to analyze the expression of SESN1, SESN2, and SESN3 at the protein level by western blot analysis. We found that neither the tested EC cell lines nor fibroblast express detectable SESN1 protein level (data not shown). Western blot analysis revealed a markedly strong expression of SESN2 in AN3CA and RL-95-2 cell line and a moderate expression in the Ishikawa cell line, but a markedly week expression in KLE and fibroblast cell line (Fig. 1a1). To avoid western blot manipulation, we decided to present in Fig. 1a1 original blot containing HEC-1-A and HEC-1-B cell lines excluded from further experiments. Western blot analysis also showed that SESN3 expression was faint in the KLE, RL-95-2, and fibroblast cell lines, while it was not detected in Ishikawa and AN3CA cell line (Fig. 1a1). The same samples were also analyzed for SESN1, SESN2, and SESN3 mRNA expression levels compared to human fibroblast cell line. Our qRT–PCR studies have revealed some discordance between mRNA and protein level as follows. In Ishikawa cell line, we observed up-regulation at mRNA level for all the analyzed SESN proteins that was not related to protein abundance revealed in western blot experiments. However, the up-regulation at SESN1 and SESN3 mRNA level was much more pronounced than protein levels, suggesting the post-transcriptional mechanisms are involved in inhibiting SESN1 and SESN3 expression in the Ishikawa cell line (Fig. 1a3, c). Up-regulated SESN2 mRNA level observed in Ishikawa cell line correlated well with protein band intensity in western blot assay (Fig. 1a3). In KLE cell line, our studies into mRNA-protein correspondence have shown poor correlation between up-regulated mRNA and SESN3 protein levels (Fig. 1b2). Moreover, qRT-PCR analysis in KLE cell line has shown down-regulated SESN1 and SESN2 that correspond to very weak protein band intensity in western blot assay, respectively (Fig. 1c, a3). In AN3CA cell line, we found a poor relationship between up-regulated mRNA level and SESN1 protein abundance revealed by the western blot assay (Fig. 1c). Moreover, our experiments indicated down-regulation of SESN3 mRNA in AN3CA cell line that correspond to a lack of protein band in the western blot assay (Fig. 1b2). In the same cell line, we observed a good correspondence for up-regulated mRNA and SESN2 protein levels shown as strong western blot fluorescence signal (Fig. 1b2). The associations between up-regulated mRNA expression in RL-95-2 cell line and protein level were attributed to SESN2 (Fig. 1b2). In RL-95-2 cell line, we observed down-regulation of mRNA SESN1 and SESN3 expression that correlate with western blot assay results only for the SESN1 protein (Fig. 1b2, c). The SESN3 protein is marked as a faint band in the western blot assay suggesting some post-transcriptional mechanisms implicated in the SESN3 protein synthesis pathway (Fig. 1a1).

### The 3′UTR of SESN1 and SESN3 genes contain a binding site for microRNA 200b/200c/429

**Fig. 2:**
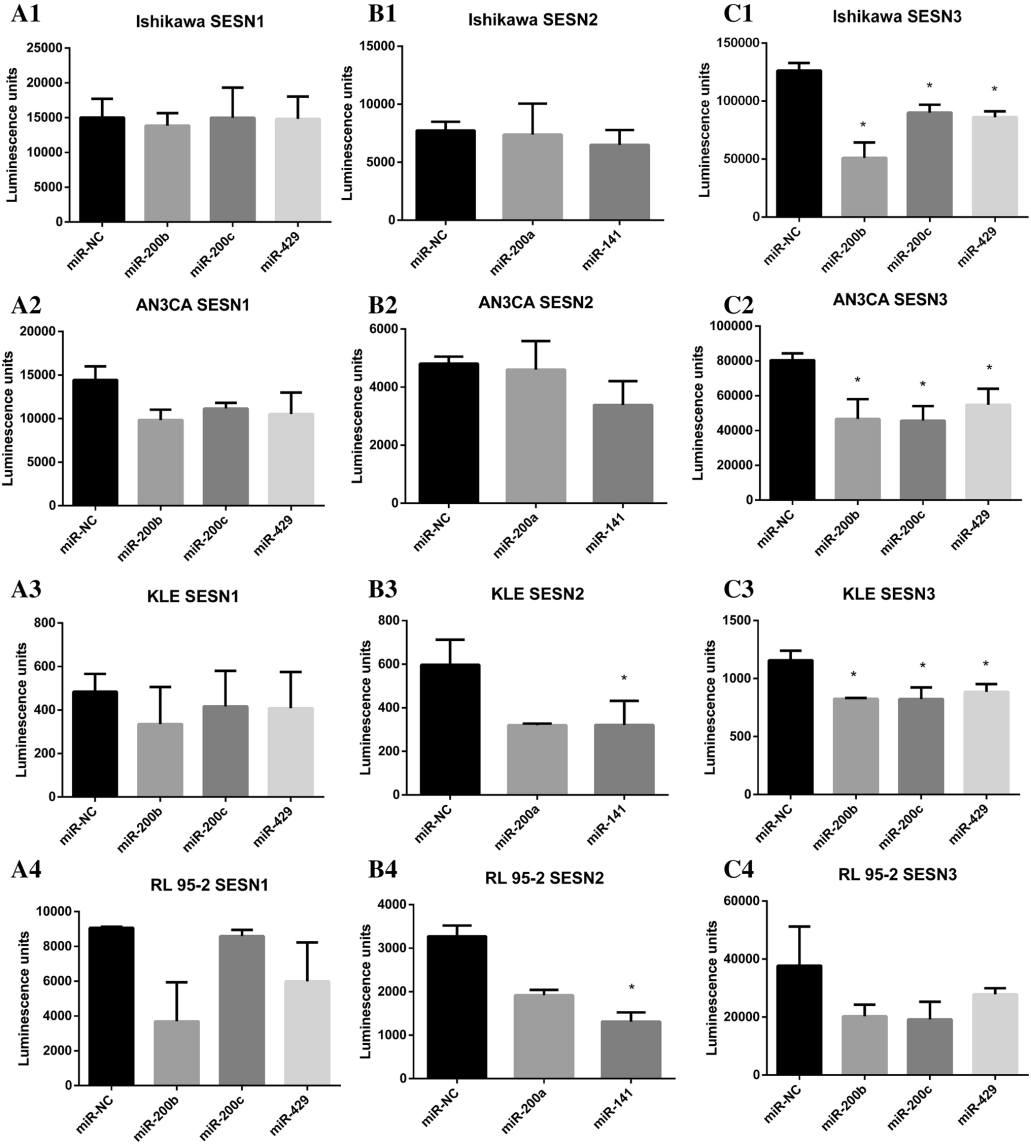
Repression of luciferase signal by the interaction between miR-200 family and the predicted 3′UTR binding site of SESN1, SESN2, and SESN3 genes. **a1**–**4** The GoClone reporter construct utilizing the RenSP luciferase gene and 3′UTR SESN1 target sequences were co-transfected with miR-200b, miR-200c, miR-429 mimics, and miR-NC into Ishikawa, AN3CA, KLE, and RL 95-2 endometrial cell lines. At 24-h post-transfection, the luciferase activity was examined. Luciferase signal from 3′UTR reporter for SESN1 gene in Ishikawa cells is reduced by 8% for miR-200b (*p* = 0.562), 1% for miR-429 (*p* = 0.758), and there is no signal change for miR-200c as compared to miR-NC. The percentage reduction in luciferase activity in AN3CA is 32 for miR-200b (*p* = 0.098), 23 for miR-200c (*p* = 0.183), and 27 for miR-429 (*p* = 0.09) as compared to miR-NC. Luciferase activity is reduced in KLE cells co-transfected with miR-200b, miR-200c, or miR-429 and SESN1 3′UTR construct as compared to miR-NC by 31% (*p* = 0.186), 14% (*p* = 0.816), and 16% (*p* = 0.523), respectively. In RL 95-2 cells, the luciferase activity is reduced by 59% for miR-200b (*p* = 0.053), 5% for miR-200c (*p* = 0.31), and 33% for miR-429 (*p* = 0.07) as compared to miR-NC. **b1**–**4** GoClone reporter construct utilizing the RenSP luciferase gene and 3′UTR SESN2 target sequences was co-transfected with miR-200a, miR-141 mimics, and miR-NC into Ishikawa, AN3CA, KLE, and RL-95-2 endometrial cell lines. At 24-h post-transfection, the luciferase activity was examined. Luciferase signal from 3′UTR reporter for SESN2 gene is reduced in the presence of miR-200a by 5% (*p* = 0.845) and in the presence of miR-141 by 16% (*p* = 0.239) as compared to miR-NC in Ishikawa cells. Luciferase signal from 3′UTR reporter for SESN2 gene is reduced in the presence of miR-200a by 4% (*p* = 0.759) and in the presence of miR-141 by 30% (*p* = 0.085) as compared to miR-NC in AN3CA cells. In KLE cells, the luciferase activity is reduced by 46% for miR-200a (*p* = 0.052) and 46% for miR-141 (*p* = 0.03) as compared to miR-NC. The co-transfection of miR-200a or miR-141 with 3′UTR SESN2 construct in RL 95-2 cells produces a substantial reduction in luciferase signal 41% for miR-200a (*p* = 0.051) and 60% for miR-141 (*p* = 0.015) as compared to miR-NC. **c1**–**4** The GoClone reporter construct utilizing the RenSP luciferase gene and 3′UTR SESN3 target sequences was co-transfected with miR-200b, miR-200c, miR-429 mimics, and miR-NC into Ishikawa, AN3CA, KLE, and RL 95-2 endometrial cell lines. At 24-h post-transfection, the luciferase activity was examined. Luciferase signal from 3′UTR reporter for SESN3 gene is significantly reduced in the presence of miR-200b, miR-200c, or miR-429 mimics as compared to miR-NC in Ishikawa, AN3CA, and KLE cell lines but not in RL 95-2 cell line. Data are shown as the mean of three replicates ± SD. *p value˂0.05

**Fig. 3:**
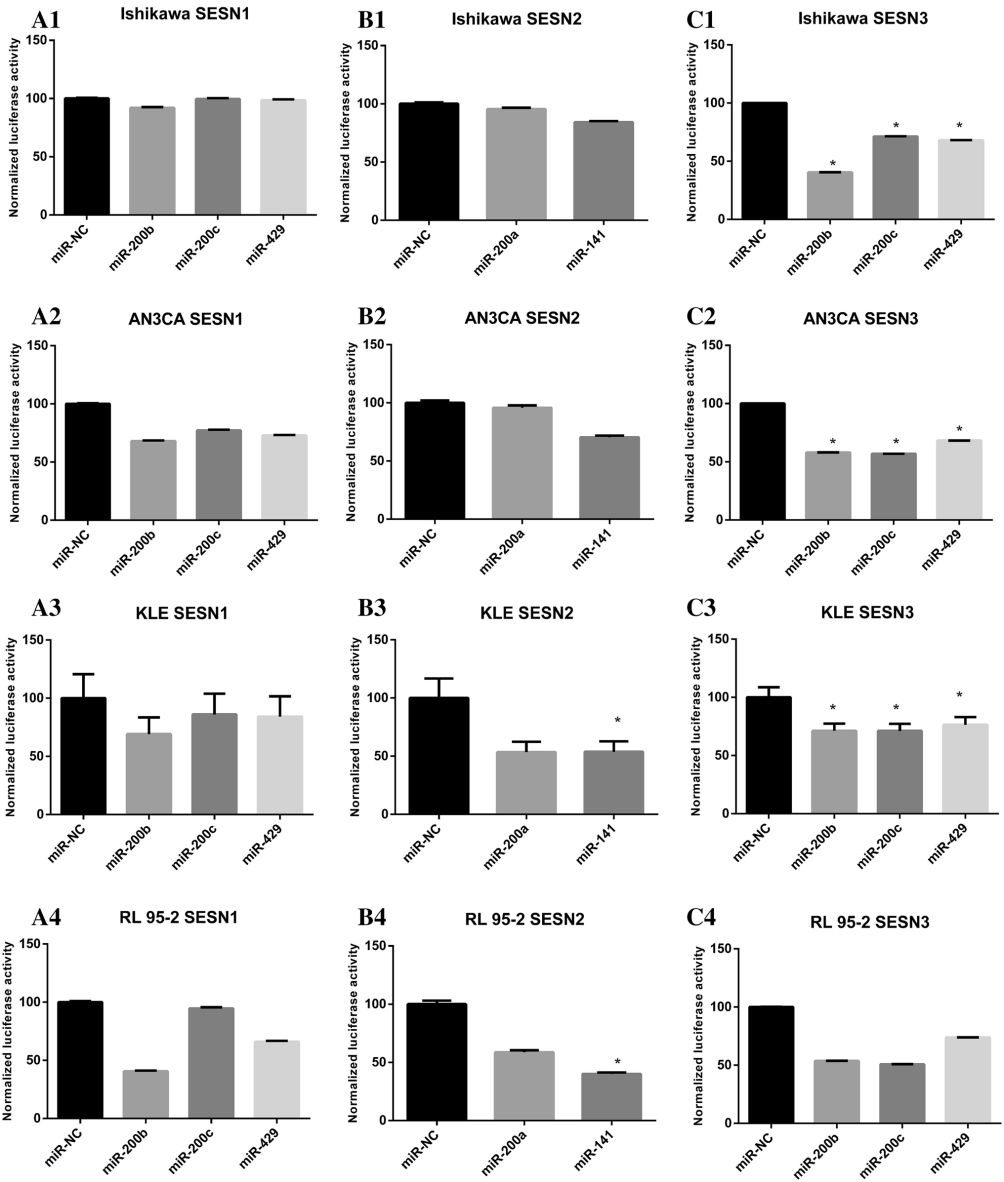
Effect of miR-200 family on the luciferase activity of GoClone reporter construct utilizing the RenSP luciferase gene and 3′UTR SESN1, SESN2, or SESN3 target sequences. The luciferase activity was normalized by comparing the effect of microRNA mimic to miR-NC (luminescence signal = specific microRNA signal/non-targeting control × 100%) and expressed as the percentage of luciferase activity. Data are shown as the mean of three replicates. *p value ˂ 0.05. **a1** In Ishikawa cells, the luciferase signal from 3′UTR reporter for SESN1 gene is reduced in the presence of miR-200b by 8%, in the presence of miR-429 by 1%, and no signal change for miR-200c as compared to miR-NC. **a2** In AN3CA cells, the luciferase signal from 3′UTR reporter for SESN1 gene is reduced in the presence of miR-200b by 32%, in the presence of miR-200c by 23%, and in the presence of miR-429 by 27% as compared to miR-NC. **a3** In KLE cells, the luciferase signal from 3′UTR reporter for SESN1 gene is reduced in the presence of miR-200b by 31%, in the presence of miR-200c by 14%, and in the presence of miR-429 by 16% as compared to miR-NC. **a4** In RL 95-2 cells, the luciferase signal from 3′UTR reporter for SESN1 gene is reduced in the presence of miR-200b by 59%, in the presence of miR-200c by 5%, and in the presence of miR-429 by 33% as compared to miR-NC. **b1** In Ishikawa cells, the luciferase signal from 3′UTR reporter for SESN2 gene is reduced in the presence of miR-200a by 5% and in the presence of miR-141 by 16% as compared to miR-NC. **b2** In AN3CA cells, the luciferase signal from 3′UTR reporter for SESN2 gene is reduced in the presence of miR-200a by 4% and in the presence of miR-141 by 30% as compared to miR-NC. **b3** In KLE cells, the luciferase signal from 3′UTR reporter for SESN2 gene is reduced in the presence of miR-200a by 46% and in the presence of miR-141 by 46% as compared to miR-NC. **b4** In RL 95-2 cells, the luciferase signal from 3′UTR reporter for SESN2 gene is reduced in the presence of miR-200a by 41% and in the presence of miR-141 by 60% as compared to miR-NC. **c1** In Ishikawa cells, the luciferase signal from 3′UTR reporter for SESN3 gene is reduced in the presence of miR-200b by 60%, in the presence of miR-200c by 29%, and in the presence of miR-429 by 32% as compared to miR-NC. **c2** In AN3CA cells, the luciferase signal from 3′UTR reporter for SESN3 gene is reduced in the presence of miR-200b by 41%, in the presence of miR-200c by 42%, and in the presence of miR-429 by 32% as compared to miR-NC. **c3** In KLE cells, the luciferase signal from 3′UTR reporter for SESN3 gene is reduced in the presence of miR-200b by 29%, in the presence of miR-200c by 29%, and in the presence of miR-429 by 24% as compared to miR-NC. **c4** In RL 95-2 cells, the luciferase signal from 3′UTR reporter for SESN3 gene is reduced in the presence of miR-200b by 46%, in the presence of miR-200c by 49%, and in the presence of miR-429 by 26% as compared to miR-NC

To identify the potential target of miR-200b, miR-200c, and miR-429, we used TargetScan program that predicts the mRNA targets of a particular microRNA. The potential interactions between 3′UTR SESN1 and SESN3 genes sequence and miR-200b, miR-200c, and miR-429 were predicted. To determine whether the selected SESN1 and SESN3 were indeed directly regulated by miR-200b, miR-200c, and miR-429, the pLightSwitch_3′UTR reporter vectors containing the 3′UTR sequence of SESN1 or SESN3 genes cloned downstream of RenSP luciferase gene were used and co-transfected with miR-200b, miR-200c, and miR-429 mimics or non-targeting control (miR-NC) into Ishikawa, AN3CA, KLE, and RL-95-2 cell lines. We observed a significantly decreased luciferase activity 24 h after the co-transfection of EC cell lines AN3CA, Ishikawa, and KLE for miR-200b, miR-200c, and miR-429 with pLightSwitch_3′UTR reporter vectors containing the 3′UTR sequence of SESN3 (Fig. 2c1–4). Ishikawa cells displayed a 60% decrease in luciferase activity for miR-200b, 29% decrease in luciferase activity for miR-200c, and 32% decrease in luciferase activity for miR-429 as compared to miR-NC (Fig. 2c1). The decrease in luciferase activity in AN3CA cell line co-transfected with pLightSwitch_3′UTR reporter vectors containing the 3′UTR sequence of SESN3 and microRNA mimics was observed for miR-200b mimics and was calculated for 41% as compared to miR-NC. In the same experimental condition, the luciferase signal reduction for co-transfection of AN3CA cell line with miR-200c and miR-429 mimics were observed at a similar level and were expressed as 42% and 32%, respectively (Fig. 2c2). KLE cells reached the comparable level of inhibition in luciferase activity for all the analyzed microRNA mimics. The percentage reduction in luciferase activity was 29 for miR-200b and miR-200c and 24 for miR-429 as compared to miR-NC (Fig. 2c3). These results indicate the potent interaction between miR-200b, miR-200c, and miR-429 and predicted SESN3 target. Luciferase activity was not significantly changed by miR-200b, miR-200c, and miR-429 in RL 95-2 endometrial cell line suggesting that SESN3 is not the direct target of miR-200b, miR-200c, and miR-429 (Fig. 2c4). However, the normalized data have shown a 46% luciferase activity reduction for miR-200b (*p* = 0.1), 49% luciferase activity reduction for miR-200c (*p* = 0.1), and 26% luciferase activity reduction for miR-429 (*p* = 0.3) as compared to miR-NC in RL 95-2 cell line co-transfected with reporter vectors containing the 3′UTR sequence of SESN3 (Fig. 3c4). The co-transfection experiments of miR-200b, miR-200c, and miR-429 mimics or miR-NC with pLightSwitch_3′UTR reporter vectors containing the 3′UTR sequence of SESN1 revealed no significant change in luciferase activity in any of the analyzed endometrial cancer cell lines (Fig. 2a1–4).

### The 3′UTR of SESN2 gene contains a binding site for microRNA 200a/141

To identify the microRNAs that regulate the SESN2, we used the computational program TargetScan. The program predicted miR-200a and miR-141 as potential regulators compatible with 3′UTR regulatory sequence of SESN2 gene. We validated whatever the predicted target could be recognized by the miR-200a and miR-141 using the pLightSwitch_3′UTR reporter vector containing the 3′UTR sequence of SESN2 gene-cloned downstream of RenSP luciferase gene. The pLightSwitch_3′UTR reporter vector was co-transfected with miR-200a and miR-141 mimics or miR-NC into Ishikawa, AN3CA, KLE, and RL-95-2 cell lines. Luciferase activity was significantly reduced 24 h after the co-transfection of endometrial cancer cell lines with pLightSwitch_3′UTR reporter vector containing the 3′UTR sequence of SESN2 gene and miR-141 mimic only in KLE and RL 95-2 cells proving efficient and stable binding to the predicted microRNA target, while no significant difference was observed for miR-200a in both the analyzed cell lines (Fig. 2b3, 4). However, the normalized data revealed the luciferase signal reduction by 46% for miR-200a in KLE cells (*p* = 0.052) and a considerably smaller reduction by 41% for miR-200a in RL 95-2 cells (*p* = 0.051) as compared to miR-NC (Fig. 3b3, 4). Luciferase activity was reduced by 5% and 16% in Ishikawa cells co-transfected with miR-200a or miR-141 and SESN2 3′UTR construct, respectively, as compared to miR-NC (Fig. 2b1). No significant differences in luciferase activity were found in regard to the co-transfection of AN3CA cells with miR-200a or miR-141 and SESN2 3′UTR construct as compared to miR-NC (Fig. 2b2). However, the normalized data revealed the luciferase signal reduction by 30% for miR-141 (*p* = 0.085) and 4% for miR-200a (*p* = 0.759) in AN3CA cells as compared to miR-NC (Fig. 3b2).

### miR-200 influences cell anoikis resistance of endometrial cancer cell lines

**Fig. 4:**
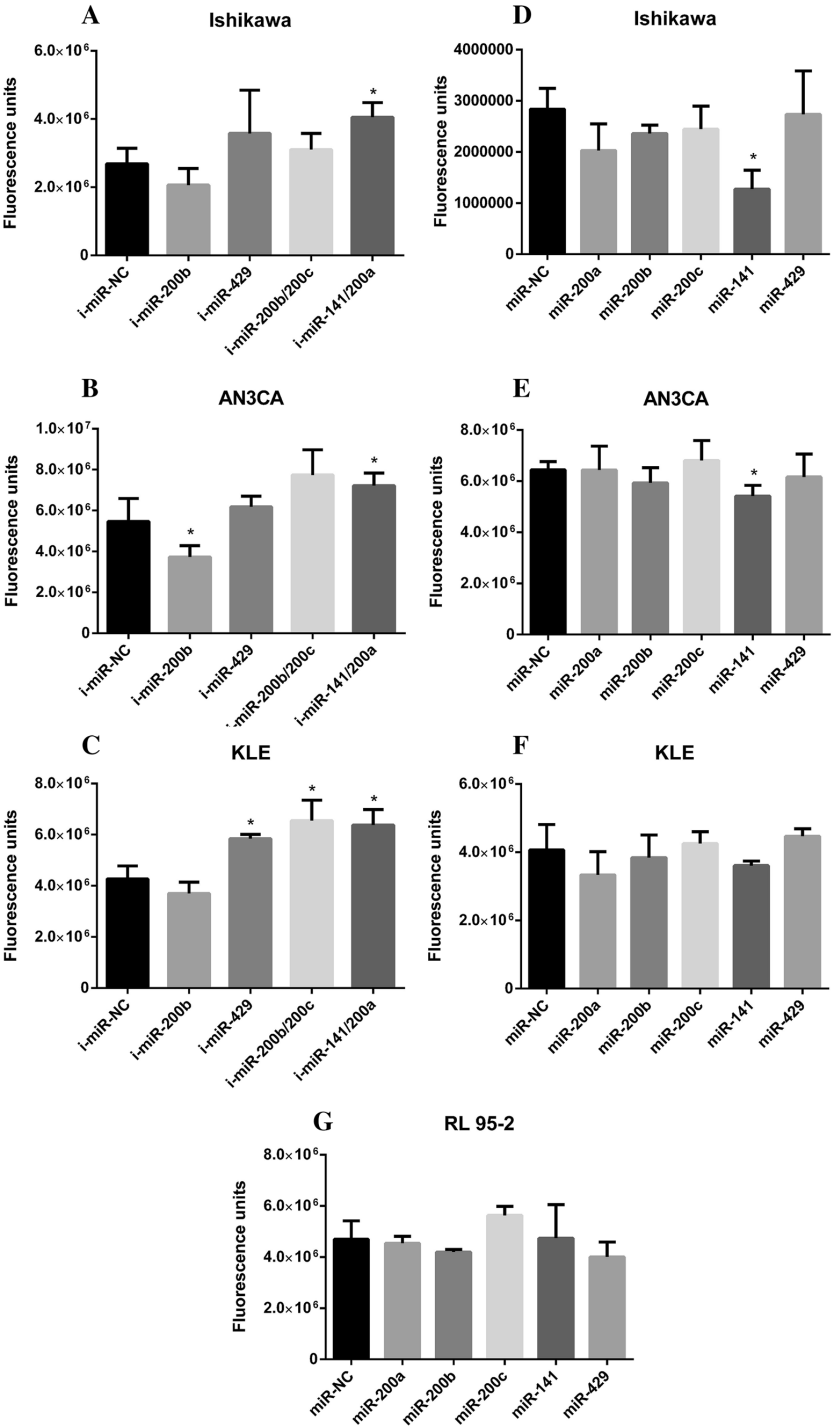
Anoikis resistance changes of endometrial cancer cell lines on exposure to miR-200 family inhibitors (**a**–**c)** or mimics (**d**–**g**). **a**–**c** Cells were seeded in an anchorage-resistant plate and transiently transfected with microRNA inhibitors i-miR-200b, i-miR-429, i-miR-200b/200c, i-miR-141/200a, or i-miR-NC. The cells were allowed to culture for 24 h. Cell viability that corresponds to anoikis resistance was determined by calcein AM. **a** In Ishikawa cells, compared to negative control, the anoikis resistance was significantly increased by i-miR-141/200a. I-miR-200b, i-miR-429, and i-miR-200b/200c have no significant effect on anoikis resistance in Ishikawa cells as compared to negative control. **b** Anoikis resistance was significantly increased by i-miR-141/200a as compared to negative control in AN3CA cells. Anoikis resistance was significantly decreased by i-miR-200b as compared to negative control in AN3CA cells. I-miR-429 and i-miR-200b/200c have no significant effect on anoikis resistance in AN3CA cells as compared to negative control. **c** Compared to negative control, the anoikis resistance was significantly increased by i-miR-429, i-miR-200b/200c, and i-miR-141/200a in KLE cells, but there was no significant effect of i-miR-200b. **d**–**g** The cells were seeded in an anchorage-resistant plate and transiently transfected with microRNA mimics miR-200a, miR-200b, miR-200c, miR-141, miR-429, or miR-NC. The cells were allowed to culture for 24 h. Cell viability that corresponds to anoikis resistance was determined by calcein AM. **d**, **e** Compared to negative control, the anoikis resistance was significantly decreased by miR-141 mimic in Ishikawa and AN3CA cell lines, but there was no significant effect of miR-200a, miR-200b, miR-200c, and miR-429 mimics. **f**, **g** In KLE and RL 95-2 cell lines, no significant differences in cell viability between the negative control and miR-200a, miR-200b, miR-200c, miR-141, miR-429 mimics were detected. All the data are presented as the mean ± SD for three experiment replicates *p < 0.05

To evaluate the impact of miR-200 family on cell anoikis resistance, the anoikis assay was performed. In this assay, the cells were plated on anchorage-resistant plate, a hydrogel coated plate that prevents cells from adhering. The cells were forced to survive under anchorage independent conditions for 24 h before being harvested for analysis. Cell viability was determined by calcein AM and showed that miR-200a/141 inhibitor cocktail significantly increases cell viability in Ishikawa, AN3CA, and KLE cells as compared to i-miR-NC (Fig. 4a–c). In KLE cells, the data demonstrated that transient transfection with miR-429 and miR-200b/200c cocktail inhibitors resulted in a significant increase in viability compared to i-miR-NC (Fig. 4c). The statistically significant reduction in viability in comparison to i-miR-NC was observed on miR-200b inhibitor transient transfection of AN3CA cells (Fig. 4b). Next, we investigated whether a correlation existed between miR-200 family mimics transient transfection and the decreased anoikis resistance expressed as decreased cells viability in endometrial cancer cell lines. We found that miR-141 mimic significantly decreases cell viability in Ishikawa and AN3CA cells but not in KLE and RL 95-2 cells compared to i-miR-NC (Fig. 4d–g). These results demonstrated that among all microRNA belonging to miR-200 family, the modulation of miR-141 expression influences anoikis resistance in endometrial cancer cells.

### The down-regulation of miR-200a and miR-141 enhanced the SESN2 expression in EC cell lines

**Fig. 5:**
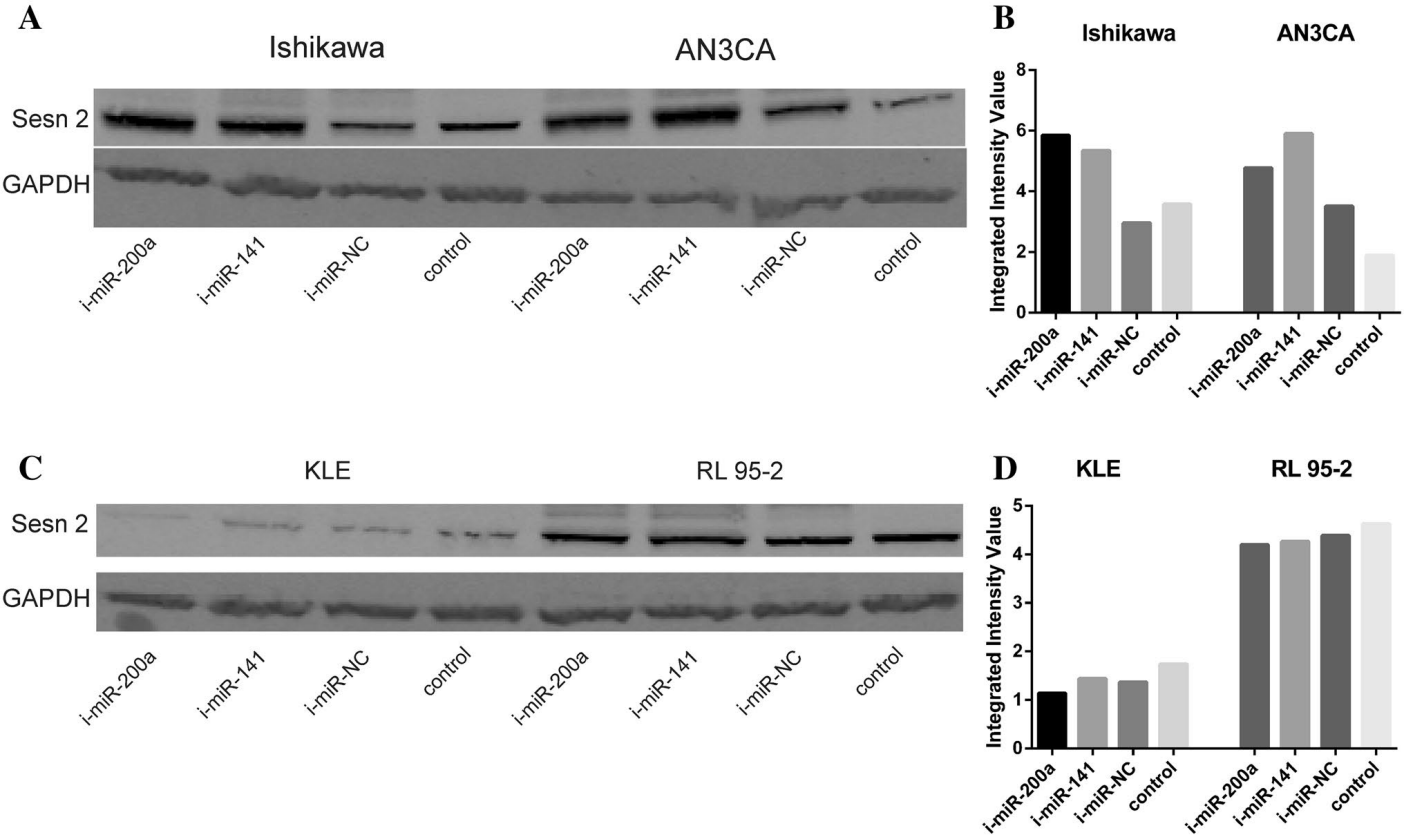
Molecular effect induced by miR-200a and miR-141 inhibitors in endometrial cancer cell lines. **a**, **b** Western blot analysis of SESN2 protein expression in Ishikawa and AN3CA cells 24 h after transient transfection with miR-200a and miR-141 inhibitors and i-miR-NC. **c**, **d** Western blot analysis of SESN2 protein expression in KLE and RL 95-2 cells 24 h after transient transfection with miR-200a and miR-141 inhibitors and i-miR-NC. The scanning with Odyssey Infrared Imaging System revealed different fluorescence intensity expressed as Integrated Intensity Value that correspond to the protein band of each analyzed inhibitor. GAPDH was used as protein loading control

To identify the potential effects of miR-200a and miR-141 on the SESN2 protein expression, miR-200a and miR-141 inhibitors were transiently transfected into Ishikawa, AN3CA, KLE, and RL 95-2 cells to downregulate the expression of endogenous miR-200a and miR-141. The western blot assay results demonstrated that 24 h after the transfection, these microRNA inhibitors enhanced the expression of SESN2 in Ishikawa and AN3CA cells compared to i-miR-NC and non-transfected cells (Fig. 5a, b). The data indicated that there were no considerable difference in SESN2 expression level in KLE and RL 95-2 cells (Fig. 5c, d). These results demonstrate that synthetic miR-200a and miR-141 inhibitors modulate the expression of SESN2 target protein.

## Discussion

Sestrins belong to conserved stress-responsive proteins regulated by various conditions including hypoxia, oxidative stress, or DNA damage [6, 10]. Physiologically, activated sestrins suppress oxidative stress by reduction of ROS level and inhibition of a mechanistic target of rapamycin complex 1 (mTORC1) signaling pathway [29]. Compelling evidence demonstrates that a dysregulation of intracellular ROS homeostasis plays an important role in the development and metastatic dissemination of cancerous cells [5, 13, 30–32]. Increasing ROS level contributes to achieving anoikis resistance of cancerous cells, which is a crucial step during metastatic colonization [5]. In the context of cancerous cells, metastasis mir-200 family members have been extensively studied [25–27, 33]. These small non-coding regulatory particles may effectively inhibit mRNA translation or cause mRNA degradation by base paring in the 3′UTR sequence of the target protein [23, 34]. In the present study, we speculated that SESN2 as well as SESN3 proteins are implicated in anoikis resistance of EC cell lines and are regulated by mir-141 and miR-200b/200c/429, respectively.

Our study is the first to show that the antioxidant protein SESN1 is not expressed in any of the analyzed EC cell lines. The expression of SESN2 protein in AN3CA cells, which were derived from undifferentiated metastatic endometrial adenocarcinoma, classified as poorly differentiated grade 3 (G3), estrogen (ER), and progesterone receptors (PR) positive type II EC [35], was higher than that in Ishikawa cells, which were derived from well-differentiated G1, type I endometrial adenocarcinoma [35]. Moreover, we observed strong expression of SESN2 protein in RL-92-5 cells derived from a moderately differentiated G2 endometrial adenosquamous carcinoma representing type I [36]. Our results showed the lowest expression of SESN2 protein in KLE cells derived from a poorly differentiated G3 endometrial tumor isolated from colon metastasis [36]. The findings suggested that expression of SESN2 protein may be correlated with tumor stage and tumor metastasis. These results are in accordance with the findings reported by Wei et al. [32]. Our study is the first to demonstrate that SESN3 expression was faint in the KLE and RL-95-2, while it was not detected in Ishikawa and AN3CA cell lines. Next, we found that SESN1 mRNA was down-regulated in KLE and RL-95-2 cell lines that is in line with the negative results obtained by western blotting. However, the SESN1 mRNA level was up-regulated in Ishikawa and AN3CA cell lines. The discordance between the presence of mRNA but lack of a protein product could be explained by mRNA turn-over and stability or post-transcriptional mechanisms involved in inhibiting the protein expression as Chen et al. postulate [37]. In our study, the enforced expression of SESN2 mRNA as well as SESN2 protein was found in three endometrial cancer cell lines Ishikawa, AN3CA, and RL-95-2 but not in KLE cell line. We also identified down-regulated SESN3 mRNA level in AN3CA and RL-95-2 that are well correlated with the western blotting results. We further found the up-regulated SESN3 mRNA level in Ishikawa and KLE cell lines that are not confirmed by the western blotting results. A lack of the integration of both mRNA and the protein expression profiles encouraged us to search for some regulatory mechanisms. Given the evidence that microRNAs effectively regulate protein expression [23, 33, 38], we used TargetScan program to predict the possible interaction between SESN1, 2, and 3 3′UTR seed sequence and microRNA. In our study, we focused on miR-200 family as a powerful regulators of epithelial-to-mesenchymal transition (EMT) and the metastasis process [23, 33]. Additionally, a miR-200 family is up-regulated in EC cell lines used in our experiments [23, 26–28, 39, 40]. However, these target predictions required an experimental validation [41]. For this purpose, the luciferase reporter assay was used, and revealed that SESN3 protein is a direct target for miR-200b/200c/429 in Ishikawa, AN3CA, and KLE cell lines. In the RL 95-2 cell line, we observed markedly (although not significantly) reduced luciferase activity for SESN3 and miR-200b/200c/429. Such phenomenon could be attributed to the attenuation of measurable response of a 3′UTR target sequence and micorRNA mimics interaction in the cell lines with a high endogenous level of particular microRNA. Our hypothesis is supported by similar findings reported by Aldred SF et al. [42]. SESN2 protein is a direct target for miR-141 in KLE and RL 95-2 cell lines. In the AN3CA cell line, we obtained strong, however not statistically important, interaction (*p* = 0.08) between SESN2 and miR-141. SESN2 and miR-200a interaction did not exert the expected attenuation of luciferase activity that was reduced by 46% (*p* = 0.052) in the KLE cell line and 24% (*p* = 0.051) in the RL 95-2 cell line. However, the lack of a significant direct interaction did not exclude SESN2 regulation by miR-200a. A similar effect was reported by Park YA et al. [39] who evaluated the effects of miR-200c on BRD7 without their direct interaction. The authors speculated the existence of another molecule that is a direct target of miR-200c and could induce the activity of BRD7 [39]. Interestingly, we observed no interaction between SESN2 3′UTR and miR-200a and miR-141 in the Ishikawa cell line and no direct interaction between SESN2 3′UTR and miR-200a in AN3CA cell line. Further, we did not find any direct interaction of miR-200b, miR-200c, and miR-429 with SESN1 3′UTR sequence in any of the analyzed EC cell lines. To the best of our knowledge, there are no available data in literature about the expression of the SESN proteins in EC cell lines as well as the regulation of SESN proteins by miR-200 family.

Our in vitro anoikis studies revealed that miR-200a/141 cocktail inhibitors enhance anoikis resistance in Ishikawa, AN3CA, and KLE cell lines. RL 95-2 cell line did not participate in this experiment. The same anoikis studies revealed that miR-141 mimic attenuates anoikis resistance in Ishikawa and AN3CA cell lines and had no effect on cell viability in KLE and RL 95-2 cell lines. Our results support the involvement of miR-141 in the regulation of anoikis resistance, which is in agreement with the hypothesis of Paoli et al. [5]. Interestingly, the down-regulation of miR-141 increased SESN2 protein expression in Ishikawa and AN3CA cell lines (no direct interaction between miR-141 and SESN2) but had no effect on SESN2 expression level in KLE and RL 95-2 cell lines. This apparent paradox might be explained by implication of another molecule that is a direct target of miR-141 and could regulate SESN2 expression. Interestingly, Mateescu et al. [22] observed that mir-141 and miR-200a directly control p38α mitogen-activated protein kinase that acts as a sensor of oxidative stress. It is therefore possible to crosstalk between miR-141 and miR-200a, p38α and SESN2 in EC cell lines. On the basis of our studies and the findings presented by Rhee et al. [16], we speculated that the possible crosstalk could be mediated in part by Nfr2, the transcription factor that is indirectly under the control of p38α and regulates the expression of SESN2. However, elucidating the detailed mechanism of the interaction between miR-141, miR-200a, p38α, Nfr2, and SESN2 would be an interesting further study.

The anoikis experiments with microRNA inhibitors revealed that i-miR-200b decreased anoikis resistance in the AN3CA cell line. We observed this same tendency with i-miR-200b in the KLE cell line, although the difference was not significant (*p* = 0.17). Moreover, we found that i-miR-429 and i-miR200b/200c cocktail increased anoikis resistance in KLE cell line. We observed this same tendency with i-miR-429 and i-miR200b/200c cocktail in AN3CA cell line, although the difference was not significant (*p* = 0.25 and *p* = 0.059, respectively). We speculated that the observed effect could be mediated by SESN3 protein, the direct target of miR-429, 200b, and 200c in AN3CA and KLE cell lines. To the best of our knowledge, the association between SESN3 and miR-200b, miR-200c, and miR-429 has not been investigated in endometrial cancer cells growth without substrate attachment. However, Nogueira et al. [19] reported that SESN3 is a major determinant of ROS level regulated by FoxO and Akt and that interacting loop has an influence on ROS-mediated apoptosis. Taken together, these preliminary experiments could warrant further studies into the role of SESN3 in the endometrial cancer anoikis mechanism.

In conclusion, the presented study for the first time showed that SESN proteins are differently expressed in four different EC cell lines. We also demonstrated that SESN proteins are the direct target for miR-200 family members, which was confirmed by luciferase reporter experiments. Our study provided new findings about the regulation of anoikis by SESN proteins. Although the significance of SESN proteins for anoikis has not been fully elucidated, we speculated that their oxidative stress activity could influence the anchorage independent growth of endometrial cancerous cells.
